# Faint, Yet Pursuing

**Published:** 1861-10

**Authors:** 


### FAINT, YET PURSUING.

WHAT a dear, delightful feeling it must be to have an humble heart. It is a thing about which a man can weep delicious tears by the hour, to feel that he is nothing—less than the least of everybody else—and yet have an eager willingness to do what he can for his Saviour.

In a moment, the crowded steamboat was on fire. Stranger and friend, maid and mistress, master and servant, dignitary and menial, were, all together, whelmed in the common disaster; some on fire, others drowning, many already dead. The only distinctions in that terrible hour were two—the ability of self-helping, and the will to help others. There was a colored man there; a planter's slave. He saved himself, and then his master; the many perished. When it was all over, the planter promptly offered the slave his freedom, and asked him what else he wanted. "Nothing; all for the love of God to-day," said the noble negro, and he must have had a heart for a king.

When we go into a house of worship on a Sabbath night, and cast our eye over the congregated thousands, we feel that they have come to hear the preacher; that is the great attraction of the evening. But while waiting for his words, a single, solitary man rises from the crowd and comes forward to the lights. Nobody knows who he is; nobody asks; nobody cares to know. All this he is aware of. Yet there is an intermediate part to be performed, comparatively trifling. He can do it, and he is willing to do it. It is a small office; yet, as it helps on the good cause, he says, Here am I; send me. He sings the tune, and falls back to make one of the undistinguished crowd again. His only talent, it may be, is to "raise the tune," and he does it. Another man is a member of some country church; his ideas seldom rise higher than the clods which his plow turns up in its furrow, and yet he knows enough to try to be a Christian. He has neither money nor influence; no gift of speech or of prayer; nor gift of conversation has he, much beyond the answer to a question by a monosyllable. What can he do without money, without influence, without gifts? Above all, what can he do for God, for that Redeemer who died for him ? He can open a door, and sweep the church, and make a fire; and he does them all, as a means of furthering the work of his Master. He takes no pay for it; claims no merit for it. His pay and claim is the privilege of doing something to help on the "good cause." We do love this doing what one may for the love of the Master. Such people must be happy. They have meat to eat which the great world knows not of. But the singer and the sexton have brothers abroad; nobler, in many respects, but in lovingness equal; men of giant minds, who have grown old and weary and physically helpless in the Master's service. We know some of them. But, like the war-horse, on a perpetual and honorable furlough, by reason of age, they answer to the trumpet's call "to arms," with the willing alacrity of youth in mind; but the body is wanting. These are the noble clergymen who, physically incapacitated from active service, are spending their time in feeding the "lambs" of the flock, and the doubting and the wayward, by writing little books, or shorter and more transient things for the magazine and the newspaper. Some of them do these things in bodily weariness and pain, which might well excuse them from the labor; but, faint, yet pursuing, they are working while they may. They can't do much; but, little as it is, it is done willingly and lovingly, in the sweet consciousness that they are working "for Jesus," not for human applause; not for money, but "for the love of God."

